# Inhibitor of DNA binding proteins revealed as orchestrators of steady state, stress and malignant hematopoiesis

**DOI:** 10.3389/fimmu.2022.934624

**Published:** 2022-08-05

**Authors:** Shweta Singh, Tanmoy Sarkar, Brad Jakubison, Stephen Gadomski, Andrew Spradlin, Kristbjorn O. Gudmundsson, Jonathan R. Keller

**Affiliations:** ^1^ Mouse Cancer Genetics Program, Center for Cancer Research, National Cancer Institute (NCI)- Frederick, Frederick, MD, United States; ^2^ Basic Science Program, Frederick National Laboratory for Cancer Research, Frederick, MD, United States

**Keywords:** ID proteins, hematopoiesis, stem cells, quiescence, stress

## Abstract

Adult mammalian hematopoiesis is a dynamic cellular process that provides a continuous supply of myeloid, lymphoid, erythroid/megakaryocyte cells for host survival. This process is sustained by regulating hematopoietic stem cells (HSCs) quiescence, proliferation and activation under homeostasis and stress, and regulating the proliferation and differentiation of downstream multipotent progenitor (MPP) and more committed progenitor cells. Inhibitor of DNA binding (ID) proteins are small helix-loop-helix (HLH) proteins that lack a basic (b) DNA binding domain present in other family members, and function as dominant-negative regulators of other bHLH proteins (E proteins) by inhibiting their transcriptional activity. ID proteins are required for normal T cell, B cell, NK and innate lymphoid cells, dendritic cell, and myeloid cell differentiation and development. However, recent evidence suggests that ID proteins are important regulators of normal and leukemic hematopoietic stem and progenitor cells (HSPCs). This chapter will review our current understanding of the function of ID proteins in HSPC development and highlight future areas of scientific investigation.

## Introduction

Hematopoiesis is sustained by a limited number of hematopoietic stem cells (HSCs) that reside in a quiescent state protected from proliferation-induced damage and exhaustion (1, 2). HSCs proliferate and differentiate to give rise to multipotent progenitor (MPP) cells with limited self-renewal potential that give rise to more restricted progenitor cells to maintain normal numbers of differentiated blood cells (3, 4). Recent evidence suggests that HSCs have a limited number of self-renewing divisions indicating that HSC quiescence and proliferation is a tightly regulated process that protects the host from HSC exhaustion and hematopoietic failure (5, 6). While HSCs are one of the best characterized vertebrate stem cells, a significant effort is still focused on understanding the molecular and cellular mechanisms that regulate HSC quiescence, self-renewal and differentiation to 1) improve methods to expand adult HSCs and their differentiated progeny for bone marrow transplantation (BMT) and cell therapies, 2) identify molecular pathways that direct adult HSC development and expansion from pluripotential stem cells, 3) improve gene editing technology in hematopoietic stem and progenitor cells (HSPCs), and 4) develop new methods to detect, prevent and treat hematologic malignancies.

Inhibitor of DNA binding proteins (ID1-4) are members of the helix-loop-helix (HLH) family of proteins that regulate cell proliferation and differentiation (7–10). ID proteins function as dominant negative regulators of other HLH proteins (E proteins) by inhibiting their DNA binding and transcriptional activity, which is essential for the differentiation and proliferation of normal tissues including muscle, nerve, lymphoid, and embryonic stem cells (11–14). ID proteins also inhibit the transcriptional activity of erythroblast transformation specific (ETS), retinoblastoma (RB), and paired box proteins (PAX) proteins, which affect cell growth and differentiation (15–17). ID proteins are critical transcriptional regulators of hematopoietic cell differentiation, and are required for the proper development of T cells, B cells, dendritic and NK and innate lymphoid cells (13, 14, 18–23). ID proteins have emerged as critical regulators of HSPC quiescence, proliferation and differentiation under homeostasis, inflammatory and genotoxic stress, and aging. These findings suggest that ID proteins could have therapeutic potential to treat myeloid proliferative neoplasia’s (MPN), myelodysplastic syndromes (MDS), and clonal hematopoiesis. Therefore, this prospective will review data related to the function of *Id* genes in regulating normal HSPC quiescence and differentiation, and their roles in hematopoietic stress and hematologic malignancies.

## Id gene expression in hematopoietic cells

*Id* gene expression in hematopoietic cells was first reported in the murine erythroleukemia cell line, MEL cells, and its expression was shown to decrease during erythroid differentiation (11, 24–26). Subsequently, *Id1* was detected in an interleukin-3 (IL-3) dependent myeloid progenitor cell line, 32Dcl3 cells, which can be induced to differentiate into neutrophils when cultured in media containing granulocyte-colony stimulating factor (G-CSF) (27). The expression of *Id1* decreased during G-CSF-induced differentiation of 32Dcl3 cells, while the binding of bHLH proteins to a canonical E-box motif increased. Furthermore, enforced expression of *Id1* blocked G-CSF-induced differentiation of 32Dcl3 cells. Taken together, these results suggested that ID1 may function during myeloid cell differentiation by disrupting bHLH protein function, the canonical target of ID proteins. Subsequent studies demonstrated that IL-3 and other myeloid growth factors (G-CSF and granulocyte/macrophage CSF, GM-CSF) induce *Id1* expression and cell proliferation in other IL-3-dependent progenitor cell lines including NFS-60 and FDC-P1 cells, while Id2 expression increases upon withdrawal of IL-3 and cell cycle arrest or differentiation (28–30). The HSC/MPP-like stem cell factor (SCF) -dependent progenitor cell line, EML cells, express ID2 but not ID1, while more committed IL-3-dependent progenitors cell lines derived from EML cells express ID1 but not ID2, suggesting ID2 may function in more primitive hematopoietic progenitor cells. Taken together, these studies demonstrated that ID1 is correlated with increased proliferation/growth and decreased differentiation of hematopoietic progenitor cell (HPC) lines, which is consistent with results from previous studies in other tissues suggesting that ID1 promotes cell proliferation and inhibits cellular differentiation (7, 9, 10, 31, 32). In addition, these studies suggest that ID2 may have functions distinct from ID1 in HSPCs.

**Figure 1:**
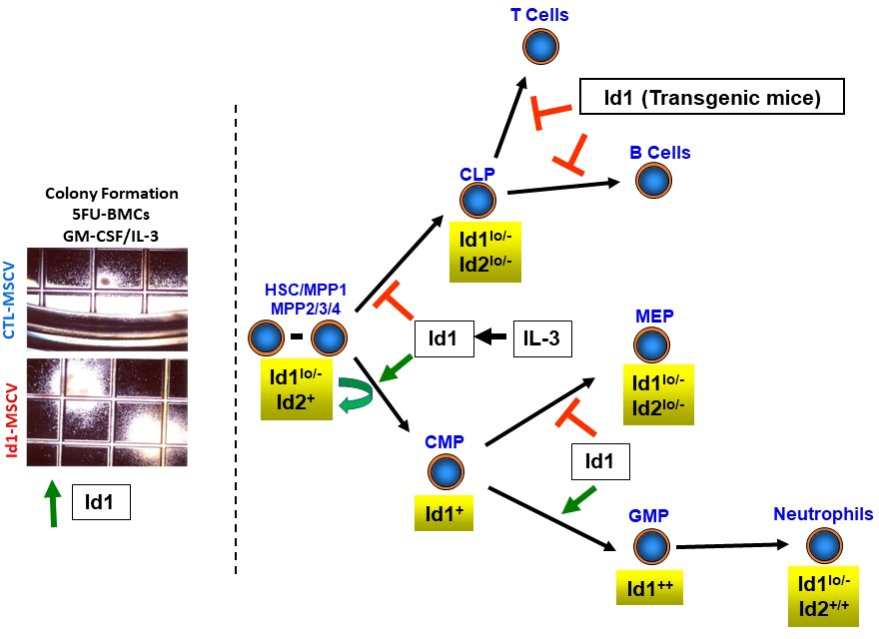
Summary of Id gene expression in hematopoietic stem and progenitor cells. Levels of *Id* gene expression are shown for hematopoietic stem cells (HSCs), multipotent progenitor (MPP) cells, clonogenic lymphoid progenitors (CLPs), common myeloid progenitors (CMPs), granulocyte/macrophage progenitors (GMPs) and megakaryocyte/erythroid progenitor cells. Photomicrographs of BMCs harvested from mice three days after the administration of 5-FU, transduced with control and *Id1* expressing retroviral vectors and cultured in methycellulose for 7-10 days (colony formation assay).

*Id1* gene expression was increased in normal murine progenitor cells during the early proliferative phase of colony formation in soft agar assays stimulated by myeloid growth factors (IL-3/SCF/IL-1/erythropoietin, EPO), while *Id2* was induced to a lesser extent and no effect on *Id3 or Id4* expression was observed in these assays (29). In comparison, *Id2* levels were increased and *Id1* levels decreased in cells from colonies that contained differentiated progeny after 7-10 days. Thus, *Id1* is expressed in normal proliferating HPCs, while *Id2* expression increases and *Id1* levels decrease as cells exit the cell cycle and differentiate into myeloid cells. Subsequent studies confirmed that myeloid hematopoietic growth factors (HGFs) (SCF, IL-3 and GM-CSF) but not lymphoid HGFs (IL-7) and erythroid/megakaryocyte HGFs (EPO/thrombopoietin, TPO) induce *Id1* expression in purified lineage-negative, Lin-/Sca-1+/c-Kit+ (LSK) BMCs that are enriched for HSCs/MPPs, suggesting that *Id1* may be required for myeloid development, but not lymphoid or erythroid development (33, 34). Analysis of *Id* gene expression in purified HSPC populations showed low levels of *Id1* expression in LSK cells and clonogenic lymphoid progenitors (CLPs), while *Id1* expression was increased in common myeloid progenitors (CMP), and further increased in more differentiated granulocyte/macrophage progenitors (GMPs), but not in megakaryocyte/erythroid progenitors (MEP), indicating a potential role for *Id1* in myeloid cell development **(** **Figure 1** **)** (33–35). Subsequent analysis of purified HSCs demonstrated that roughly 5-10% of normal HSCs defined as LSK/Flk2-/CD150+/CD48- express *Id1* during steady state hematopoiesis, and that myeloid HGFs induce *Id1* in HSCs (36, 37). While the expression of *Id1* increases in committed myeloid progenitor cells (CMP and GMP), the expression of *Id1* decreases during the final stages of neutrophil maturation. Specifically, FACS purified normal neutrophils express low levels of ID1 protein compared to Lin- cells that are comprised of LSK cells and more committed Lin-/cKit+/Sca-1- (LK) progenitor cells that include CMPs/GMPs (33). ID1 expression decreases during G-CSF- and GM-CSF-induced neutrophil differentiation of myeloid progenitor cell lines, 32Dcl3 and MPRO, respectively (27, 33). In contrast, mature neutrophils in *Id1^EGFP^* reporter mice show high levels of ID1/EGFP expression compared to CMP’s and GMP’s, suggesting that neutrophils express high levels of ID1 (34, 36). However, EGFP might not accurately reflect ID1 protein levels in neutrophils since ID proteins are rapidly degraded and the stability of EGFP and Id1 may differ significantly in these terminally differentiated cells. Future studies are needed to define the precise expression and function of ID1 and other ID genes in maturing neutrophils and macrophages.

*ID2* expression is 3-fold higher than *ID1* and 6.5-fold higher than *ID3* in purified human cord blood (CB) HSCs suggesting that human HSCs express high levels of ID2 (38). Little or no expression of ID1 was detected in purified CD34+38- human bone marrow HSPCs, but was induced by myeloid HGFs including GM-CSF/IL-3, but not SCF/EPO (erythroid) and SCF/TPO (megakaryocytes) HGFs (39). Thus, ID1 is expressed at low levels in human HSPCs and is induced by myeloid HGFs, and is consistent with results obtained with murine bone marrow cells (BMCs). Similarly, ID1 protein expression is induced during the early proliferative phase of normal human neutrophil/eosinophil differentiation in culture; after which, ID1 expression rapidly declines, while ID2 steadily increases (40). Furthermore, gain and loss of function studies showed that ID2 expression promotes eosinophil/neutrophil differentiation, and ID1 expression promotes neutrophil differentiation of human CD34+ progenitor cells *in vitro* and *in vivo* (40). Collectively, these results demonstrate that ID1 functions during the early proliferative stages of myeloid development, while ID2 may be required for the final stages of myeloid cell development. Further studies are needed to more precisely define, 1) the cell-specific and temporal expression of *Id1* and *Id2*, 2) how *Id1* and *Id2* gene expression is regulated, and 3) their molecular mechanism(s) of action during myeloid cell differentiation in normal human and murine cells.

## Insights into *Id* gene function in hematopoietic stem and progenitor cells through the lens of enforced expression

Initial studies to define the physiological function of *Id* genes in hematopoietic cells *in vivo* demonstrated that transgenic mice overexpressing *Id1* or *Id2* during T cell differentiation manifest a severe block in T cell development **(** **Figure 1** **) (** 41, 42). Similarly, transgenic mice that overexpress *Id1* during B cell differentiation show impaired B cell development (43). These studies are consistent with the strict requirement for E2A homodimers in B cell development and E2A homo and heterodimers in T cell development, and the dominant negative regulation of E protein function by ID proteins (20, 44).

To study *Id* gene function in HSPCs, *Id* genes were overexpressed in normal murine HSPCs using retroviral vectors and analyzed for growth and differentiation *in vitro* (33). Enforced expression of *Id1* in HSPCs resulted in a significant increase in the size and number of colonies in soft agar in the presence of SCF, IL-3 and GM-CSF **(** **Figure 1** **)** (39). Furthermore, these colonies contained increased numbers of primitive and differentiating myeloid cells, suggesting that *Id1* promotes myeloid cell hyperplasia without blocking differentiation *in vitro*. However, immortalized hematopoietic progenitor cell (HPC) lines that escape normal senescence and differentiation could be readily derived from BMCs overexpressing *Id1* in liquid culture containing SCF (39). The HPC lines resembled CMP-like progenitors by immunophenotype and lineage specific gene expression, and continued to divide in SCF; however, these cells were not arrested in differentiation and retained the ability to differentiate in response to G-CSF and GM-CSF *in vitro*. In other studies, overexpression of *Id1* in purified LSK cells inhibited B cell development and promoted myeloid/dendritic cell development in cultures supplemented with SCF/FLT3/IL-7 (34). Furthermore, LSK/Flk2- and lymphoid-primed multipotent progenitor (LMPP) cells isolated from mice that overexpress a dominant form of E protein (ET2) that prevents ID protein binding, showed increased B cell, and reduced myeloid cell development when cultured *in vitro*. Collectively these studies demonstrate that *Id1* promotes the proliferation of murine HSPCs and myeloid development, while inhibiting lymphoid development *in vitro*.

*Id1*-overexpressing HSPCs showed increased myeloid and decreased B cell repopulation potential when transplanted into γ-irradiated (IR) recipients *in vivo* **(** **Figure 1** **) (** 33, 39). In addition, thymocyte repopulation was inhibited during the early stages of T cell development after transplantation; however, no effect on T cell repopulation was observed in PBCs 4-6 months after BMT. A comparison of Id1/EGFP expression in repopulating T cells and myeloid cells in the same mouse by flow cytometry showed a significant reduction in Id1/EGFP expression in T cells compared to myeloid cells. Thus, it was reasoned that T cells expressing high levels of *Id1* do not repopulate, while low levels of *Id1* expression are permissive for T cell development *in vivo*, and account for the recovered T cell development over time. Historically, murine BMCs cultured in HGFs cocktails that included IL-3 to promote HSC cycling and increase retroviral vector transduction, show significantly reduced lymphoid repopulation when transplanted *in vivo*. It is tempting to speculate that inhibition of lymphoid cell repopulation in these BMT experiments was due to the induction of *Id1* by IL-3. In this regard, HGFs that promote *Id1* expression and inhibit lymphoid development of HSCs should be avoided in HSC expansion media. Finally, mice transplanted with *Id1*-overexpressing HSPCs become moribund within a year after transplantation and develop a myeloid proliferative neoplasia (MPN) including 1) increased myeloid blasts in BM, 2) splenomegaly and extramedullary hematopoiesis (myeloid and erythroid), and 3) myeloid cell infiltration in liver, which does not progress to acute myeloid leukemia (AML) (39). Collectively, enforced expression of *Id1* regulates HSPC fate by promoting myeloid and inhibiting lymphoid development *in vitro* and *in vivo*, and *Id1* can promote HSPC proliferation and immortalize HSPCs *in vitro*, and promote a myeloproliferative disease *in vivo*.

HPC lines were also derived from murine BMCs transduced with lentiviral vectors expressing inducible *Id2* (Id2-HPCs), which were established in conditions that support primitive B cell growth including SCF, FLT-3 and IL-7 on S17 stromal cells (45). The Id2-HPC lines express c-Kit, CD43 and low levels of B220, but lack the expression of other cell surface markers expressed on more mature myeloid and lymphoid lineage cells. Id2-HPC lines are multipotential and differentiate into myeloid cells in the presence of IL-3, GM-CSF, and M-CSF *in vitro* and *in vivo*, under conditions that maintain *Id2* expression. Id2-HPCs differentiate into lymphoid cells (B cell and T cell) upon withdrawal of ID2 *in vitro* and *in vivo* consistent with the ability of ID2 to inhibit E protein function. The lymphoid potential of the Id2-HPC lines was lost with cell passaging suggesting that the Id2-HPC lines may undergo further myeloid differentiation and commitment *in vitro*. It is possible that the Id2-HPC lines that have lost lymphoid potential may resemble the SCF-dependent CMP-like HPC lines that overexpress *Id1* (39). The Id2-HPC line transcriptome resembles the transcriptome of the previously described *E2a^-/-^* multipotential cell lines (pre-pro-B cells) that were established in SCF, FLT-3, IL-7 and stromal cells (45), which is consistent with the ability of ID2 to inhibit the function of E2A and prevent lymphoid development. In comparison, over expression of *Id2* in human cord blood CD34+ HSPCs resulted in expansion of CD34+/CD38-/CD90+/CD45RA- HSCs, and a skewing toward myeloid (CMP/MEP) versus lymphoid (B/NK/GMP) by immunophenotype analysis *in vitro*, and reduced lymphoid development when transplanted into NSG mice (38). HSPCs isolated from NSG mice transplanted with ID2-overexpressing CB-HSPCs show increased expression of stem cell genes and genes expressed in primitive myeloid differentiation programs, and decreased expression of B cell factors, *EBF1* and *FOXO1*, which are E2A target genes (46). ID2-mediated expansion of HSCs was rescued by overexpressing E2A *in vitro*, and overexpression of E2A promoted lymphoid development *in vivo*. Knock down of the *E2A* target gene, *EBF1*, which is required for lymphoid development, resulted in an increase in HSC numbers. These results suggested that ID2 expands or maintains HSC numbers by inhibiting lymphoid lineage priming, which leads to a reduction in HSC numbers (38). Taken together, overexpression of *ID2* expands HSCs and restrains B cell development in human HSPCs. These studies suggest that ID2 may function to regulate human and mouse HSCs function *in vivo.*

Hematopoietic progenitor cell lines were also established by overexpressing *Id3* in fetal liver cells and BMC cultures containing SCF, FLT-3, IL-7, and stromal cells, and resemble pre-pro B cells (Id3-HPC lines) (47). Id3-HPC lines are multipotential and retain myeloid, B cell and T cell potential *in vitro* and *in vivo*. The lymphoid potential of Id3-HPC lines was induced by down regulation of *Id3* expression in developing B cells *in vivo*, supporting previous studies that high levels of *Id3* impair B and T cell development (48–51). Secondary transplantation of BMCs from primary BMT recipients that received Id3-HPCs showed residual myeloid reconstitution, but no lymphoid reconstitution suggesting that Id3-HPCs are not HSC/MPP-like cells (47). Id3-HPC lines could not be established from human CB HSPCs; however, *Id3* overexpressing CB HSPCs cells showed enhanced proliferation *in vitro* and limited repopulation potential in NSG mice. Additional experiments are required to further understand the mechanism(s) of *Id3*-mediated immortalization in murine and human HSPCs compared to *Id1* and *Id2* immortalization.

## Hematopoietic phenotypes in mouse models of *Id* gene loss of function

### Conventional *Id1^-/-^* mice

The first *Id1*- deficient (*Id1^-/-^*) mouse model was generated by gene targeting, which replaced the first exon and part of the promoter of *Id1* with a neomycin resistance gene cassette. Conventional *Id1^-/-^* mice were born at normal mendelian frequencies, were fertile and showed normal lifespan with no overt abnormalities (52). No significant difference in the number of mature hematopoietic cells was reported in PBCs, BMCs and spleen cells of *Id1^-/-^* mice, suggesting that *Id1* is not required for normal hematopoietic development. However, a more detailed analysis of these mice revealed that *Id1^-/-^* mice have impaired hematopoietic development including 1) increased myeloid cells and decreased lymphoid cells in PBCs, 2) decreased BM cellularity, 3) decreased numbers of LSK cells, 4) decreased numbers of LSK/CD34-/Flk2- cells enriched for HSCs, 5) increased cycling of LSK cells, and 6) increased proliferation and differentiation of LSK cells *in vitro* (35). No difference in the repopulation potential of *Id1^-/-^* and *Id1^+/+^* BMCs was observed in primary BMT recipients; however, *Id1^-/-^* BMCs showed impaired secondary repopulation ability albeit with low statistical significance, suggesting that *Id1^-/-^* HSCs have a defect in self-renewal. Collectively, these investigators concluded that *Id1^-/-^* HSCs show increased cycling and increased myeloid commitment, which resulted in decreased HSC self-renewal in secondary BMT recipients, suggesting that *Id1* is required to maintain HSCs.

A second *Id1^-/-^* mouse model was generated by inserting the EGFP coding sequence downstream of the Id1 transcriptional start site, which results in an *Id1* null allele (*Id1^EGFP/EGFP^*) (37). In comparison to the Jankovic et al. *Id1^-/-^* mouse model described above, the hematopoietic phenotypes observed in *Id1^EGFP/EGFP^* mice included decreased numbers of HSCs (LSK/CD150+/CD48-); however, this report showed no effect on the cycling of HSC enriched populations, no difference in the development of mature lymphoid and myeloid cell populations in BMCs or PBCs. *Id1^EGFP/EGFP^* mice showed no difference in repopulation in primary BMT recipients, but reduced secondary repopulation potential suggesting that *Id1* was required for HSC development and maintenance. However, it should be noted that the secondary BMT was performed 16 days after primary BMT, which is a significant deviation from the typical secondary BMT protocol, which is usually performed 10-16 weeks after primary BMT, when hematopoiesis resembles more steady-state conditions (37). Therefore, it is difficult to conclude from these studies if *Id1* is required for HSC self-renewal.

A third study also analyzed hematopoietic development in the *Id1^-/-^* mouse model used in the Jankovic et al. study discussed above (35) and confirmed that 1) myeloid cells were increased and lymphoid cells decreased in BMCs and PBCs, 2) BM cellularity was decreased, and 3) LSK cycling was increased (53). This study showed no difference in the number of HSC-enriched cells (LSK/CD34- cells) and HSC function *in vivo*; however, the two studies used different cell surface antigens to immunophenotype the HSCs, which could explain the differences in HSC numbers. Finally, the two studies agreed and showed no difference in HSC repopulation potential in primary BMT recipient mice and differed in serial repopulation potential leaving open the question whether *Id1* functions in HSC self-renewal.

Based on the known function of *Id1* in other cellular contexts, it might be predicted that loss of *Id1* would lead to reduced cell cycling and increased B cell development in the hematopoietic compartment of *Id1^-/-^* mice. However, *Id1^-/-^* mice show the opposite hematopoietic phenotypes including increased LSK cycling and proliferation, and decreased B cell development (35, 53). Since *Id1* expression is ablated in all cells in conventional *Id1^-/-^* mice, and *Id* genes are widely expressed in other tissues including endothelial cells (ECs) and skeletal stem cells (SSCs) and their progeny, which are cellular constituents of the hematopoietic microenvironment (HME) (8, 54–57), it is possible that loss of *Id1* function in the HME could contribute to the hematopoietic phenotypes observed in conventional *Id1^-/-^*. Therefore, in the third study, *Id1^+/+^* and *Id1^-/-^* BMCs were transplanted into γ-IR *Id1^-/-^* or *Id1^+/+^* recipient mice and monitored for hematopoietic development (53). *Id1^+/+^* BMCs transplanted into *Id1^-/-^* recipients showed impaired hematopoietic development similar to the hematopoietic phenotypes observed in the conventional *Id1^-/-^* mice including decreased BM cellularity, increased myeloid and decreased erythroid development. Importantly, *Id1^-/-^* BMCs showed normal hematopoietic development when transplanted into *Id1^+/+^* recipient mice. These results were confirmed in a recent study using a mouse model that lacked *Id1* and *Id3* expression in the HME, since HME phenotype was less severe in mice on a pure C57BL/6 background and *Id3* can compensate for loss of *Id1* in some models (58). Transplantation of normal BMCs into γ-IR *Id1* ^-/-^ *;Id3* ^-/-^ recipient mice showed a significant decrease in BM cellularity, decreased B cell development, increased HSC cycling and decreased HSC numbers (56). Finally, *Id1^-/-^* stromal cells show altered cytokine production *in vitro*, and cytokine levels were deregulated in conventional *Id1^-/-^* mice *in vivo*. Collectively, these results demonstrated that *Id1* is required for the proper function of the HME, and that the hematopoietic phenotypes observed in conventional *Id1^-/-^* mice could, in part, be explained by the loss of *Id1* function in the HME.

### Conditional loss of *Id1* in endothelial cells

Endothelial cells are critical cellular components of the HME and are required to maintain steady state hematopoiesis (59–63). SECs are critical cellular components of the HME and are required to maintain steady state hematopoiesis, in part, through angiocrine signaling (64, 65). Lineage tracing studies using vascular endothelial cadherin (VE-Cad) transgenic mice *Cdh5(PAC)-CreERT2* bred to *Rosa26-mT/mG* mice showed that transition endothelial vessels (H vessels), proliferate to regenerate diaphyseal sinusoidal ECs (SECs) within forty days under homeostasis (66). Recent evidence suggest that *Id* genes promote EC proliferation and vessel regeneration under stress (67–69); thus, it was hypothesized that *Id* genes may be required for proper HME function by maintaining ECs under steady state conditions and stress (56). Therefore, *Cdh5(PAC)-CreERT2* mice were bred to *Id1^F/F^;Id3^-/-^* mice to specifically ablate *Id1* and *Id3* expression in ECs, since *Id1* and *Id3* are required for proper HME in mice on a pure C57/BL6 background. Loss of sinusoidal integrity was observed in *Id1^-/-^; Id3^-/-^* mice characterized by dilated, leaky, and apoptotic BM SECs that increased in severity over time (56). The proliferation of *Id1^-/-^; Id3^-/-^* SECs, and transition endothelial vessels was significantly reduced *in vitro* and *in vivo*, leading to impaired vascular integrity under steady state conditions, which was more severe following acute stress. The disruption in sinusoidal integrity and neovascularization in *Id1^-/-^;Id3^-/-^* mice led to a progressive decline in hematopoiesis, marked by increased HSC activation, proliferation, differentiation, migration, and exhaustion. Thus, *Id1* and *Id3* are required for the survival and steady state regeneration of BM SECs, which provide a supportive niche for HSC quiescence and survival. Future studies are needed to examine if *Id* genes regulate other cells in the HME including subtypes of SSCs (Leptin-cre+, Nestin-ER-cre+, and NG2-cre+), which functionally support hematopoiesis, and their downstream progeny including osteoblasts and chondrocytes (60, 63, 70). Future studies are needed to examine Id gene function in the neural niche, since nerve fibers such as adrenergic and cholinergic nerves are instructive for hematopoietic mobilization and quiescence respectively.

### Conventional *Id3^-/-^* mice

*Id3^-/-^* mice are born at normal mendelian frequencies, are fertile, and young mice show no overt phenotypes. *Id3^-/-^* mice have normal numbers of developing B cells, but show impaired humoral immunity, B-cell proliferation, and develop a unique autoimmune disease, Sjogren’s syndrome, and γδ-T cell hyperplasia with age (49, 50, 71, 72). In addition, *Id3^-/-^* mice show severely impaired positive and negative thymocyte selection (50). Transplantation of *Id3^-/-^* BMCs into γ-IR mice show normal myeloid and B cell repopulation, but impaired T cell repopulation in secondary recipient mice, suggesting that *Id3^-/-^* is not required for HSC maintenance (37). However, further studies are needed to examine if *Id3* is required for HSC self-renewal in serial BMT assays separated by 10-12 weeks (37). In other studies, no difference in donor B cells, T cells, neutrophils, HSCs and MPP repopulation were observed in mice 12 weeks after competitive BMT of *Id1^-/-^Id3^-/-^* BMCs compared to controls, confirming that *Id3* is not required for HSC repopulation of primary BMT recipient mice; however, HSC self-renewal was not evaluated in these assays (56). Therefore, additional studies are needed to examine the requirement of *Id3* in HSC development.

### Conventional *Id2^-/-^* mice

The majority of conventional *Id2^-/-^* mice show perinatal lethality and are born at less than ten percent of the normal mendelian frequencies. The surviving mice lack Langerhans cells, splenic dendritic cells, and NK cells, and show absence of lymph nodes and Peyer’s patches, which demonstrates the requirement for ID2 in the development of these cells (18, 22). Surviving *Id2^-/-^* mice also show increased B cell development, decreased erythroid cells and no effect on myeloid cell development (73). Gain and loss of *Id2* function studies in normal HSPCs confirmed that *Id2* intrinsically inhibits B cell development *in vivo* by negatively regulating E2A. In addition, these studies showed that ID2 binds to PU.1 and interferes with PU.1’s ability to inhibit GATA-1 transcriptional activity, suggesting a potential mechanism of action for how ID2 promotes erythroid development (73). Additional experiments are needed to further explore this mechanism of action, and more precisely define the progenitors that express *Id2*, and where cell fate is determined and how the expression of *Id2* is regulated. In this regard, *Gfi-1* has been identified as a direct transcriptional repressor of *Id2*, and high levels of *Id2* are expressed in *Gfi-1* ^-/-^ BMCs, and these mice show defects in B cell, T cell, and neutrophil development (74, 75). In addition, these mice have impaired short term reconstituting cell (STRC) activity or ability to radio-protect lethally irradiated recipient mice, and have significantly reduced numbers of HSCs (74–76). Reducing *Id2* levels in *Gfi-1* ^-/-^ mice (*Gfi-1* ^-/-^;*Id2^+/-^* mice) partially restores B cell development by overcoming the block in B cell development at the pro-B cell stage, and reduces myeloid hyperplasia, but does not rescue T cell development. These results provide evidence for a direct link between *Gfi-1* and the B cell transcriptional network *via Id2*, which inhibits E2A function required for B cell development (76). While reducing the levels of *Id2* in *Gfi-1* ^-/-^ mice rescued the myeloid hyperplasia in the spleen, it did not rescue neutrophil differentiation. Additional studies are needed to uncover how *Id2* promotes myeloid expansion in *Gfi-1* ^-/-^ mice and why normal myeloid development is not restored. Finally, reducing *Id2* levels in *Gfi-1* ^-/-^ mice partially restores the number of STRCs, CMP and MEP progenitors and differentiating erythroid cells in the BM bone marrow, which is sufficient to radio-protect lethally irradiated BMT recipient mice (77). Increased red cell production in *Gfi-1* ^-/-^;*Id2^+/-^* mice was correlated with increased expression of *Gata1*, *Eklf* and *EpoR*, which are required for erythroid development. It was proposed that ID2 inhibits E2A/Scl complexes that regulate erythroid gene expression *via* a multiprotein transcription factor complex that binds paired E-box/GATA sites in the promoters of *Gata1, Eklf and EpoR*. However; the precise molecular mechanism(s) of *Id2* action that rescue the erythroid lineage in *Gfi-1* ^-/-^ mice remain to be defined. Finally, reducing *Id2* levels in *Gfi-1* ^-/-^ mice does not increase HSC numbers and rescue the defect in HSC function in competitive repopulation assays indicating that other genes mediate HSC loss in *Gfi-1* ^-/-^ mice. Collectively, these observations suggest that *Id2* regulates HSPC fate at multiple cellular levels, but leaves open the question whether *Id2* regulates HSC development.

### Conditional *Id2* ^-/-^ mice

**Figure 2:**
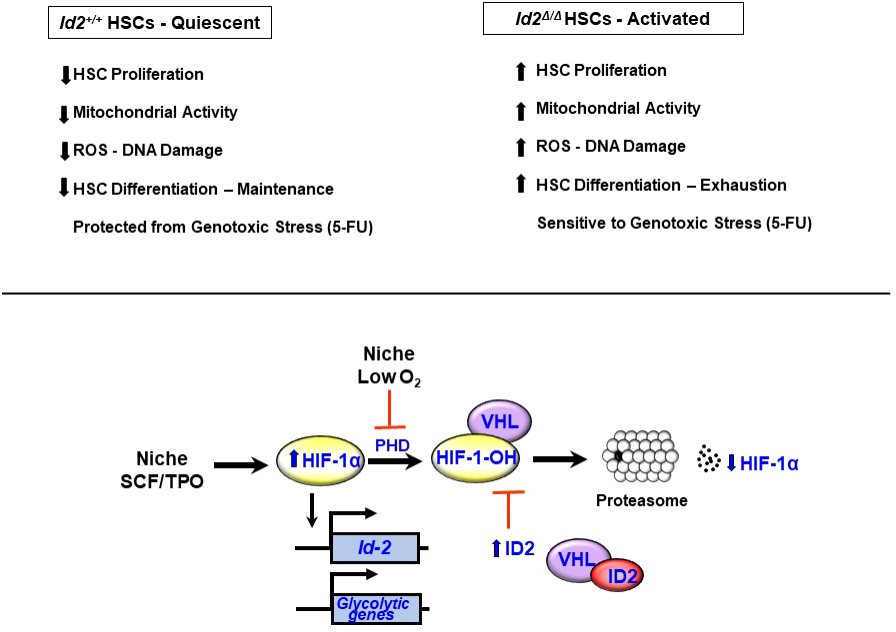
*Id2* is required for HSC quiescence and maintenance. Summary of hematopoietic phenotypes in *Id2* ablated mice. *Hif-1α* expression is induced in HSCs by stem cell factor (SCF) and thombopoietin (TPO). HIF-1α protein levels are maintained at low levels under normoxic conditions *via* the action of proline hydroxylases (PHDs), which hydroxylate HIF-1α and promotes its association with the VHL complex, ubiquitination and proteasomal degradation. Under low O_2_ conditions, PHD is inhibited resulting in reduced levels of hydroxylated HIF-1α, and reduced ubiquitination and stabilization of HIF-1α. ID2 also acts to stabilize HIF-1α by binding to VHL, which prevents ubiquination and proteosomal degradation of HIF-1α and promotes HSC quiescence.

**Figure 3:**
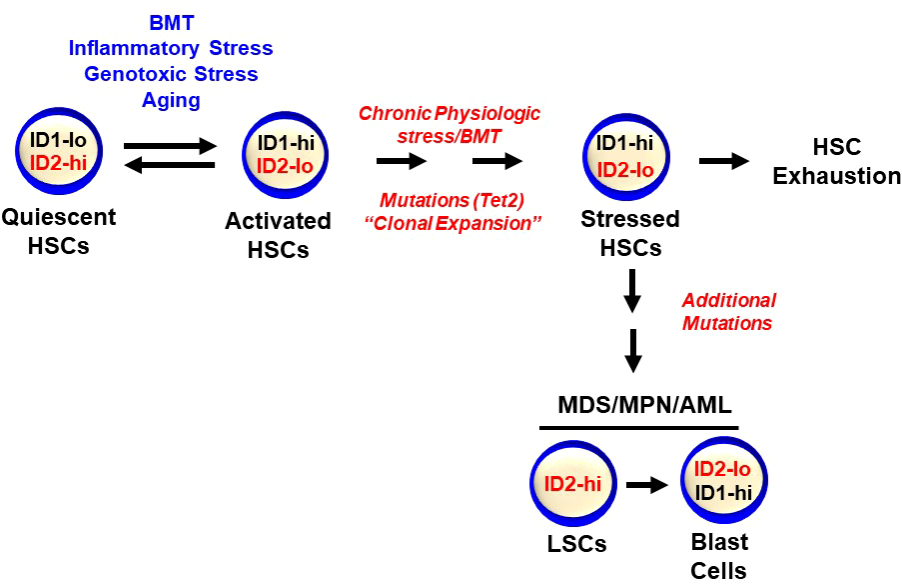
*Id1* and *Id2* are differentially expressed during HSC activation and quiescence. HSCs express low levels of *Id1* that are induced during stress including BMT, genotoxic and inflammatory stress, and aging. *Id2* is required to maintain HSC quiescence and *Id2* decreases during HSC proliferation and activation, after which, *Id1* levels decrease and *Id2* levels increase as HSCs return to quiescence. Chronic stress can lead to HSC exhaustion and clonal hematopoiesis and hematopoietic malignancies.

Few conventional *Id2^-/-^* mice survive beyond birth suggesting that the surviving *Id2^-/-^* mice may have compensated in some way to promote the survival of *Id2^-/-^* mice. Therefore, the intrinsic requirement of *Id2* in HSCs was evaluated in conditional *Id2^F/F^* mice. Specifically, BMCs from *Mx1-cre;Id2^F/F^* mice (78) were transplanted in γ-IR recipient mice (chimeric mice) to reconstitute the host hematopoietic system and eliminate any contribution that loss of *Id2* function might have in the HME. Chimeric mice were treated with *pIpC* to induce interferon production and ablate *Id2* expression in hematopoietic cells six weeks after BMT, and then examined for hematopoietic development after ten weeks. Chimeric mice showed a significant reduction in the total number immunophenotypic HSCs, and decreased donor reconstitution of competitively transplanted primary recipient mice confirming that *Id2* is required to maintain HSCs in chimeric mice **(** **Figure 2** **)**. Furthermore, *Id2^-/-^* chimeric mice showed reduced overall survival due to anemia and BM failure compared to *Id2^+/+^* chimeric mice indicating that ID2 is intrinsically required for HSC maintenance. Mechanistically, *Id2^-/-^* HSCs showed increased proliferation and cycling, mitochondrial activation, reactive oxygen species (ROS) production and differentiation *in vitro* and *in vivo*. Pathway analysis of differentially expressed genes in *Id2^+/+^* and *Id2^-/-^* HSCs revealed increased expression of genes that regulate cellular proliferation and genes that regulate oxidative phosphorylation in *Id2^-/-^* HSCs compared to *Id2^+/+^* HSCs. In addition, gene set expression analysis (GSEA) and pathway analysis revealed that HIF-1α target genes were decreased *Id2^-/-^* HSCs suggesting that ID2 might affect the levels or function of HIF-1α. In this regard, *HIF-1α^-/-^* mice show similar hematopoietic phenotypes with *Id2^-/-^* mice including increased cycling, decreased quiescence, and increased susceptibility to 5-FU treatment (79). HIF-1α protein levels were reduced in purified *Id2^-/-^* HSCs, and loss of HSC function in *Id2^-/-^* mice could be restored by chemically stabilizing HIF-1α and overexpression of stabilized HIF-1α *in vitro* and *in vivo*. Mechanistically, ID2 stabilizes HIF-1α by binding to VHL and interfering with HIF-1α ubiquitination and proteasomal degradation **(** **Figure 2** **)**. Collectively, ID2 is required to maintain HSC quiescence, a function that is distinct from ID1, which promotes HSC proliferation. *Id2* and *Id1* expression are inversely correlated during hematopoietic stress, where *Id2* expression decreases in HSCs as they exit quiescence and *Id1* expression increases; after which, *Id1* decreases and *Id2* increases when HSCs resume quiescence **(** **Figure 3** **)**. Future experiments are needed to determine if *Id2* regulates quiescence of leukemic stem cells (LSCs), and if there are additional mechanism(s) of *Id2* action in normal and LSCs.

## ID1 and stress hematopoiesis

**Figure 4:**
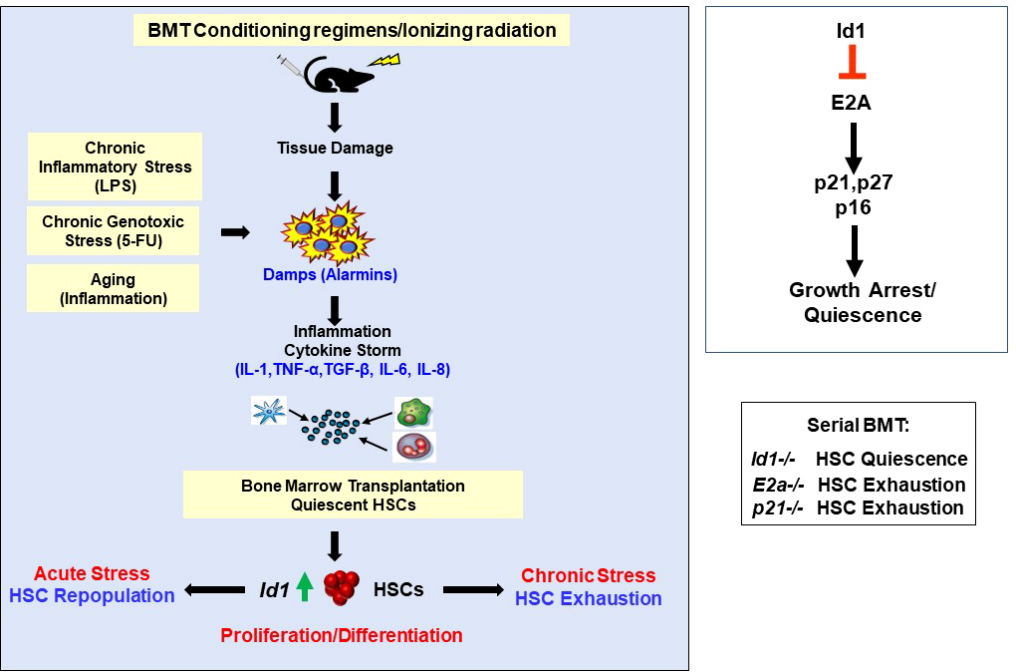
Ablation of Id1 Protects HSCs from chronic proliferative stress including BMT, chronic genotoxic and inflammatory stress, and aging. Summary of mechanism of ID1 action in HSCs, and gene knockout mouse models in the molecular pathway.

*Id* genes are early response genes that are induced in NIH-3T3 fibroblasts by serum and growth factors, and are required for re-entry of serum-starved cells into cell cycle, demonstrating the requirement for *Id* genes in HGF-induced proliferation (stress) (80). *Id* genes are expressed at low levels in most adult tissues, and are induced by a wide array of extracellular signals in response to stress or injury to promote tissue repair and regeneration (10, 67, 68). HSCs express low levels of *Id1* under steady state conditions, and *Id1* is induced by HGFs that promote myeloid proliferation and differentiation including IL-3 (33, 34). Furthermore, enforced expression of *Id1* in HSPCs promotes myeloid cell proliferation at the expense of lymphoid development, suggesting that ID1 may function during hematopoietic stress (33, 39). Therefore, to evaluate the role of *Id1* in hematopoietic stress, *Id1^-/-^* BMCs were serially transplanted in γ-IR recipient mice. No significant difference in the repopulation potential of *Id1^-/-^* BMCs compared to *Id1^-/-^* BMCs was observed in primary BMT recipients. However, *Id1^-/-^* BMCs showed enhanced self-renewal potential and promoted the survival of serially transplanted mice significantly beyond the potential of *Id1^+/+^* BMCs **(** **Figure 4** **) (** 36). Increased numbers of HSCs were detected in mice serially transplanted with *Id1^-/-^* BMCs compared to *Id1^+/+^* BMCs, demonstrating that *Id1^-/-^* HSCs are maintained and protected from exhaustion during chronic stress. Furthermore, *Id1^-/-^* HSCs in serially transplanted mice showed reduced cycling, DNA damage (γ-H2AX phosphorylation), mitochondrial biogenesis and activation, and ROS levels compared to *Id1^+/+^* HSCs, indicating that *Id1^-/-^* HSCs show increased quiescence during stress (36). Comparative transcriptome analysis of purified donor *Id1^-/-^* and *Id1^+/+^* HSCs confirmed that *Id1^-/-^* HSCs have an increased quiescent molecular signature including reduced expression of genes involved in cell cycle, oxidative phosphorylation, ribosomal biogenesis, and protein synthesis. Taken together, *Id1^-/-^* HSCs show increased quiescence and reduced proliferation and activation during hematopoietic stress compared to *Id1^+/+^* HSCs.

BMT conditioning regimens including γ-IR damages the HME resulting in acute and chronic inflammation due to the production of alarmins, recruitment of inflammatory cells and the production of pro-inflammatory cytokines (cytokine storm) (81–85). Initial reports demonstrated that myeloid HGFs induce *Id1* expression and proliferation of HSPCs *in vitro* (33, 34). Subsequent studies showed that *Id1* can be induced in HSCs by a variety of proinflammatory cytokines *in vitro*, and HSCs that express *Id1* are actively proliferating (36). In addition, these studies found that *Id1* is induced in HSCs by proinflammatory cytokines *in vivo*, and in HSCs after 6 Gy γ-IR. Thus, during chronic stress *Id1^-/-^* HSCs fail to properly respond to cytokine-induced proliferation and differentiation and are protected from exhaustion *in vitro and vivo* during serial BMT.

It is difficult to predict which cytokines in the cytokine storm induce *Id1* in HSCs after BMT *in vivo*; however, many cytokines signal *via* the Jak/Stat pathway suggesting that JAK inhibitors could protect HSCs from chronic proliferative stress. In this regard, JAK1 is an intracellular tyrosine kinase signaling molecule required for HSCs to respond to stress cytokines including IFN-α/β/γ and IL-3 (86). *Jak1* deficient HSCs exhibited increased quiescence, inability to enter cell cycle, reduced response to type I interferons and IL-3, and impaired ability to reconstitute hematopoiesis during BMT and stress (86). Since ID1 proteins are induced by IL-3 in normal HSPCs and cell lines (33), the increased quiescence of *Jak1^-/-^* HSCs during stress may be mediated, in part, by reduced expression of *Id1*. Therefore, mice were treated with small molecule inhibitors of the JAK/STAT pathway after BMT to inhibit *Id1* induction in HSCs. JAK/STAT inhibitors partially inhibited the induction of *Id1* in HSCs after BMT in γ-IR recipient mice demonstrating that pro-inflammatory cytokines produced after γ-IR induce *Id1* in HSCs *in vivo* (36). Future studies could target other relevant ligands (cytokines), receptors, and downstream signaling pathways involved in proinflammatory cytokine signaling during BMT including IL-6, IL-1, tumor necrosis factor (TNF), IL-8 and others to prevent HSC exhaustion and improve BMT.

It was hypothesized that since *Id1^-/-^* HSCs are protected from chronic proliferative stress and exhaustion during serial BMT, ablation of *Id1* expression in HSPCs during other conditions of chronic stress including inflammatory and genotoxic stress, and aging could prevent HSC exhaustion **(** **Figure 4** **)**. Indeed, studies found that *Id1^-/-^* HSCs were also preserved in models of chronic proliferative stress including chronic inflammatory stress (LPS) and genotoxic stress (5-FU) (36, 87). In addition, HSCs from aged (2 years old) *Id1^-/-^* mice resemble more youthful HSCs including, 1) an immunophenotype of young HSCs, 2) increased HSC function in serial BMT assays, 3) increased HSCs in G0, 4) decreased HSC DNA damage, 4) reduced HSC ROS levels, and 5) reduced HSC mitochondrial stress compared to *Id1^+/+^* HSCs. Future experiments are needed to further define the molecular mechanism(s) that protect *Id1^-/-^* HSCs from aging. Collectively, these results demonstrate that *Id1^-/-^* HSCs are more quiescent and resistant to chronic proliferative stress including inflammatory and genotoxic stress and aging. Clonal hematopoiesis, MPN and MDS are associated with increased inflammation (84, 88–90), which could induce *Id1*, promote HSPC proliferation during clonal hematopoiesis, and increase mutational load and genomic instability over time.

## Mechanism(s) of Id1 action in hematopoietic stem and progenitor cell growth and quiescence

*Id1^-/-^* HSCs fail to properly respond to cytokine-induced proliferation and differentiation and show decreased BrdU incorporation and cell cycling after stress *in vitro* and *in vivo* (36). Since E proteins inhibit cell proliferation, in part, by promoting the expression of cyclin dependent kinase inhibitors (Cdki’s) (91–94), and ID proteins inhibit E-proteins resulting in increased proliferation, it is reasonable to hypothesize that the reduced cycling of *Id1^-/-^* HSCs is due to unrestrained E protein transcriptional activity and increased Cdki expression (Id1-E2a-Cdki pathway) **(** **Figure 4** **)**. Increased *p21* expression was observed in *Id1^-/-^* LSK cells compared to *Id1^+/+^* HSCs (35). Subsequent reports demonstrated that *Id1^-/-^* HSCs show increased *p27* and *p16* expression and reduced proliferation in expansion cultures compared to *Id1^+/+^* HSCs, and *Id1^-/-^* HSCs show increased expression of *p21* and *p27* after 14 weeks in competitive BMT assays compared to *Id1^+/+^* HSCs (36). Furthermore, knock down of *E2a* and *p16* expression in *Id1^-/-^* HSPC expansion cultures increased HSC proliferation suggesting that *Id1* increases HSC proliferation, in part, by restraining E protein function and reducing *p16* expression (36). Importantly, *E2a^-/-^* HSCs show increased cycling, decreased serial repopulation potential, and decreased expression of *p21* and *p27* expression (95–97), and *p21^-/-^* HSCs have decreased serial repopulation populations potential (98). Thus, *Id1^-/-^* HSCs demonstrate opposite hematopoietic phenotypes to *E2a^-/-^* and *p21^-/-^* HSCs, whereby *Id1^-/-^* HSCs show increased quiescence and are protected from hematopoietic stress and exhaustion, and *E2a^-/-^* and *p21^-/-^* HSCs show increased proliferation and activation and are sensitive to hematopoietic stress and premature exhaustion. Thus, the Id-E-Cdki pathway critically regulates HSC cycling during stress. In other studies, the E protein(E47) – Cdki (p21) pathway was shown to be important in preventing HSC exhaustion during BMT and 5-FU mediated stress (99). Specifically, genetic experiments examining the requirement for E47- p21 pathway in maintaining HSCs under stress showed that *E47^het^p21^het^* HSCs exhibit decreased serial BMT repopulation when compared to *E47^het^p21^WT^*, which have increased p21 levels. The E-Cdki pathway is conserved in endothelial cells (ECs), where *p21* and *p27* RNA expression is increased in ECs that lack *Id* gene expression, and growth inhibition of *Id1^-/-^Id3^-/-^* ECs *in vitro* was rescued by knocking down *E2-2* expression (56). In comparison, *E2a* and *p16* shRNAs partially restore the proliferation of *Id1^-/-^* HSCs *in vitro*, suggesting that other E and Cdki proteins may regulate quiescence in *Id1* ablated HSCs, or *Id1* regulates HSC functions through other target genes and pathways. Therefore, it will be important to evaluate if knock down of other E proteins, *E2-2* and *Heb*, and other Cdki’s rescue *Id1^-/-^* HSC proliferation *in vitro*. In this regard, the role of *E2-2* and *Heb* in HSC development in conditional mouse models has not been evaluated. Furthermore, it would be important to know if reducing the expression of *p21* and other Cdki’s *via* genetic experiments can rescue the hematopoietic phenotypes in *Id1^-/-^* mice *in vivo*. Finally, additional transcriptomic, proteomic, and single cell analysis of normal, stressed, or aged *Id1^-/-^* HSCs might uncover additional molecular pathways that regulate HSC quiescence.

## Id Genes and hematopoietic malignancies

*Id* gene expression has been correlated with the initiation, progression, and metastasis of cancer in many tissues (7, 10, 100). *Id* genes are frequently overexpressed in advanced stage chronic myeloid leukemia (CML), AML and MDS patient samples while low or no levels of ID1 were detected in normal CD34+ HSPCs (39, 101–110). Knock down of *ID1* expression in the AML cell line, MO-7e, resulted in decreased growth and suggested that some AML cells may require ID1 for growth. Furthermore, analysis of *ID* gene expression in an AML patient cohort showed that high levels of *ID* gene expression were correlated with *FLT3-ITD, RAS, EVI-1* and *C/EBPA* mutations, suggesting *ID* gene expression may be induced downstream of oncogene activated signal transduction pathways (111). *ID1* was identified as a common downstream target of oncogenic tyrosine kinases including FLT3-ITD and BCR-ABL (112). Stable KD of *ID1* in K562 (BCR-ABL) and Molm14 (FLT3-ITD) AML cell lines inhibited their growth *in vitro* confirming that *ID1* is required for the growth of some AML cell lines. Analysis of *Id1* expression in a 237 AML patient cohort demonstrated that AML patients with high levels of *ID1* gene expression were less likely to achieve complete remission and were correlated with shorter disease-free survival and overall survival (113). Thus, it was suggested that *ID1* expression levels may provide a molecular tool for refining risk classification of AML. In addition, these studies suggest that Id1 may represent a candidate for targeted therapy to treat AMLs (106).

To explore the intrinsic role of *Id* genes in hematopoietic malignancies, *Id1*-overexpressing HSPCs were transplanted into lethally irradiated recipient mice and monitored for survival. *Id1*-overexpressing mice died roughly one year after BMT compared to control mice, and sick mice showed 1) myeloid/erythroid cell hyperplasia and increased numbers of immature cells in the BM, 2) splenomegaly and extramedullary hematopoiesis, and 3) peripheral blood monocytosis, indicating that these mice succumbed to a MPN that did not progress to AML (39). *ID1* expression is increased in leukemic cells from AML patients with t (8, 21) translocations, and AML1-ETO regulates the *ID1* promoter, suggesting a role for *ID1* in AML1-ETO leukemia (114). Ablation of *Id1* in AML1-ETO transduced murine fetal liver cells delays the onset and development of leukemia by roughly 130 days after transplantation *in vivo* (115). Furthermore, conditional loss of *Id1* in established AML-ETO-leukemia’s slows the development of leukemia and promotes animal survival, suggesting that *Id1* is also required for the maintenance of leukemia. Mechanistically, these studies provided evidence that *Id1* promotes AML-ETO leukemic cell growth by interacting with Akt and increasing its activity. In other studies, the progression of leukemia was significantly delayed after transplantation of MLL-AF9-transduced *Id1^-/-^* FL cells compared to MLL-AF9-transduced *Id1^+/+^* fetal liver cells (116). The delay in *Id1^-/-^* MLL-AF9 leukemia was reversed in FL cells that lack *p21*, which is consistent with previous studies demonstrating that ID proteins promote HSPC proliferation by inhibiting the E-Cdki pathway. Interestingly, loss of *Id1* accelerated the progression of MLL-AF9-induced leukemia of BMCs, suggesting that *Id1* is not required for MLL-AF9-induced leukemia of BMCs, and that leukemogenesis may differ significantly for FL and BM HSPCs. The cellular and molecular mechanism(s) that account for the differences in the progression of leukemia in these models is not currently known. Taken together, these studies provide evidence that *ID1* is required for the initiation and progression of oncogene driven leukemias. Future studies are needed to determine if *ID1* is required for AML cell line growth in xenotransplantation and human AML PDX models, and if the recently identified inhibitors of ID1 including cannabidiol and AGX51 show any therapeutic benefit in these models (117–119). Since MPN and MDS and myeloid malignancies that develop with age are strongly correlated with inflammation and *Id1* is induced in HSPCs downstream of pro-inflammatory signals, future studies are needed to better understand the role of *ID1* in these diseases and the molecular mechanism(s) of ID1 action (84, 89, 120).

Ablation of *Id2* in HSCs results in increased proliferation and HSC activation suggesting that ID2 may function as a tumor suppressor. Interestingly, Ko et al. showed that mice transplanted with *Id2^-/-^* fetal liver cells develop leukocytosis after 6 months that resembles a myeloproliferative disorder, and that over expression of ID2 delays the onset *BCR-ABL*-induced CML *in vivo* (121). In addition, loss of *Id2* expression is associated with increased MLL-AF9-induced leukemia in mice, and over expression of *Id2* inhibits the growth of MV4-11 and MOLM-13 AML cell lines that express MLL-AF9, and Kasumi AML cells that express AML-ETO (122). Together, these results suggest that ID2 may function as a tumor suppressor in hematopoietic malignancies. In addition, mice that lack *Id2* develop intestinal adenomas, and show a hyperproliferation of colon stem cells during embryonic development due to increased Wnt/B-catenin signaling, suggesting that ID2 may function as a tumor suppressor in other cell types (123, 124). Analysis of ID2 expression in 145 AML patient BMCs showed that AML patient cells with high levels of ID2 expression were correlated with lower complete remission and shorter overall survival, and was a predictor of poor chemotherapy response (103). Analysis of *ID2* expression in a subset of MLL-rearranged AML patient cells indicated that MLL patients (35 patients) with high levels of *ID2* expression had a significantly better overall and event free survival than patients with low levels of *ID2* (122). Further studies are needed to examine if levels of *ID2* expression are prognostic for AML patient subsets, and to determine if ID2 is expressed in LSCs and functions to regulate their quiescence and survival **(** **Figure 3** **)**.

## Conclusion and perspectives

ID proteins have emerged as important regulators of HSPC quiescence (ID2) and proliferation (ID1). Specifically, low levels of ID1 are expressed in primitive HSPCs, but are induced in HSPC after acute stress including BMT, inflammatory and genotoxic stress, and promote HSPC proliferation and myeloid development, while inhibiting lymphoid development **(** **Figure 3** **)**. Upon resolution of an acute stress, HSCs return to quiescence with low levels of ID1 and hematopoiesis resumes under steady state conditions. However, under chronic proliferative stress ID1 levels remain high and HSCs undergo excessive proliferation, exhaustion and bone marrow failure. Reducing ID1 levels during serial BMT, chronic inflammatory and genotoxic stress and aging may be therapeutic to protect HSCs from exhaustion. In addition, since hematopoietic malignancies and bone marrow failure syndromes are often accompanied by inflammation and increased ID1 expression, reducing ID1 levels could be therapeutic by reducing preleukemic proliferation and clonal expansion, which could delay the onset and reduce the incidence of hematopoietic malignancies and bone marrow failure syndromes. ID2 has emerged as a critical regulator of normal HSC quiescence and shows opposite expression to ID1, where ID2 levels decrease as HSCs exit quiescence and ID1 levels increase during cell proliferation. Maintaining high levels of ID2 *in vitro* and *in vivo* could be exploited to expand HSCs for gene and cell therapies including BMT. Opposing expression of ID1 and ID2 is also observed during the final stages of myeloid development, where ID1 expression is increased in myeloid progenitors (CMP/GMP), and then decreases as cells exit the cell cycle and differentiate, while ID2 expression is increased in mature neutrophils. Furthermore, current evidence suggests that ID1 and ID2 may function during neutrophil and eosinophil development, however, gain and loss of function studies are needed to reveal if these genes are required for the differentiation and function of these cells. Since ID2 regulates normal HSC quiescence, the potential role of ID2 in LSC quiescence and resistance to current therapies remains to be explored.

## Author contributions

SS, TS, BJ, SG, AS, KG, and JK contributed to writing and editing the review and figures. All authors contributed to the article and approved the submitted version.

## Funding

This project has been funded in part with federal funds from the Frederick National Laboratory for Cancer Research, NIH (HHSN261200800001E). The content of this publication does not necessarily reflect the views or policies of the Department of Health and Human Services, nor does the mention of trade names, commercial products, or organizations imply endorsements by the US Government.

## Conflict of interest

The authors declare that the research was conducted in the absence of any commercial or financial relationships that could be construed as a potential conflict of interest.

## Publisher’s note

All claims expressed in this article are solely those of the authors and do not necessarily represent those of their affiliated organizations, or those of the publisher, the editors and the reviewers. Any product that may be evaluated in this article, or claim that may be made by its manufacturer, is not guaranteed or endorsed by the publisher.

## References

[1] Trumpp A Essers M Wilson A. Awakening dormant haematopoietic stem cells. Nat Rev Immunol (2010) 10(3):201–9. doi: 10.1038/nri2726 20182459

[2] Pinho S Frenette PS. Haematopoietic stem cell activity and interactions with the niche. Nat Rev Mol Cell Biol (2019) 20(5):303–20. doi: 10.1038/s41580-019-0103-9 PMC6483843 30745579

[3] Seita J Weissman IL. Hematopoietic stem cell: self-renewal versus differentiation. Wiley Interdiscip Rev Syst Biol Med (2010) 2(6):640–53. doi: 10.1002/wsbm.86 PMC2950323 20890962

[4] Ito K Frenette PS. HSC contribution in making steady-state blood. Immunity (2016) 45(3):464–6. doi: 10.1016/j.immuni.2016.09.002 PMC5048748 27653597

[5] Bernitz JM Kim HS MacArthur B Sieburg H Moore K. Hematopoietic stem cells count and remember self-renewal divisions. Cell (2016) 167(5):1296–309.e10. doi: 10.1016/j.cell.2016.10.022 27839867 PMC5115957

[6] Qiu J Papatsenko D Niu X Schaniel C Moore K. Divisional history and hematopoietic stem cell function during homeostasis. Stem Cell Rep (2014) 2(4):473–90. doi: 10.1016/j.stemcr.2014.01.016 PMC3986626 24749072

[7] Roschger C Cabrele C. The id-protein family in developmental and cancer-associated pathways. Cell Commun Signal (2017) 15(1):7. doi: 10.1186/s12964-016-0161-y 28122577 PMC5267474

[8] Wang LH Baker NE. E proteins and id proteins: helix-loop-helix partners in development and disease. Dev Cell (2015) 35(3):269–80. doi: 10.1016/j.devcel.2015.10.019 PMC4684411 26555048

[9] Norton JD. ID Helix-loop-helix proteins in cell growth, differentiation and tumorigenesis. J Cell Sci (2000) 113(Pt 22):3897–905. doi: 10.1242/jcs.113.22.3897 11058077

[10] Lasorella A Benezra R Iavarone A. The ID proteins: master regulators of cancer stem cells and tumour aggressiveness. Nat Rev Cancer (2014) 14(2):77–91. doi: 10.1038/nrc3638 24442143

[11] Benezra R Davis RL Lockshon D Turner DL Weintraub H. The protein id: a negative regulator of helix-loop-helix DNA binding proteins. Cell (1990) 61(1):49–59. doi: 10.1016/0092-8674(90)90214-Y 2156629

[12] Murre C. Helix-loop-helix proteins and lymphocyte development. Nat Immunol (2005) 6(11):1079–86. doi: 10.1038/ni1260 16239924

[13] de Pooter RF Kee BL. E proteins and the regulation of early lymphocyte development. Immunol Rev (2010) 238(1):93–109. doi: 10.1111/j.1600-065X.2010.00957.x 20969587 PMC2992984

[14] Engel I Murre C. The function of e- and id proteins in lymphocyte development. Nat Rev Immunol (2001) 1(3):193–9. doi: 10.1038/35105060 11905828

[15] Ohtani N Zebedee Z Huot TJ Stinson JA Sugimoto M Ohashi Y. Opposing effects of ets and id proteins on p16INK4a expression during cellular senescence. Nature (2001) 409(6823):1067–70. doi: 10.1038/35059131 11234019

[16] Lasorella A Noseda M Beyna M Yokota Y Iavarone A. Id2 is a retinoblastoma protein target and mediates signalling by myc oncoproteins. Nature (2000) 407(6804):592–8. doi: 10.1038/35036504 11034201

[17] Roberts EC Deed RW Inoue T Norton JD Sharrocks AD. Id helix-loop-helix proteins antagonize pax transcription factor activity by inhibiting DNA binding. Mol Cell Biol (2001) 21(2):524–33. doi: 10.1128/MCB.21.2.524-533.2001 PMC86614 11134340

[18] Hacker C Kirsch RD Ju XS Hieronymus T Gust TC Kuhl C. Transcriptional profiling identifies Id2 function in dendritic cell development. Nat Immunol (2003) 4(4):380–6. doi: 10.1038/ni903 12598895

[19] Jackson JT Hu Y Liu R Masson F D’Amico A Carotta S. Id2 expression delineates differential checkpoints in the genetic program of CD8alpha+ and CD103+ dendritic cell lineages. EMBO J (2011) 30(13):2690–704. doi: 10.1038/emboj.2011.163 PMC3155298 21587207

[20] Kee BL E. And ID proteins branch out. Nat Rev Immunol (2009) 9(3):175–84. doi: 10.1038/nri2507 19240756

[21] Kee BL Quong MW Murre C. E2A proteins: essential regulators at multiple stages of b-cell development. Immunol Rev (2000) 175:138–49. doi: 10.1111/j.1600-065X.2000.imr017514.x 10933599

[22] Yokota Y Mansouri A Mori S Sugawara S Adachi S Nishikawa S. Development of peripheral lymphoid organs and natural killer cells depends on the helix-loop-helix inhibitor Id2. Nature (1999) 397(6721):702–6. doi: 10.1038/17812 10067894

[23] Boos MD Yokota Y Eberl G Kee BL. Mature natural killer cell and lymphoid tissue-inducing cell development requires Id2-mediated suppression of e protein activity. J Exp Med (2007) 204(5):1119–30. doi: 10.1084/jem.20061959 PMC2118569 17452521

[24] Sun XH Copeland NG Jenkins NA Baltimore D. Id proteins Id1 and Id2 selectively inhibit DNA binding by one class of helix-loop-helix proteins. Mol Cell Biol (1991) 11(11):5603–11. 10.1128/mcb.11.11.5603-5611.1991 PMC361931 1922066

[25] Lister J Forrester WC Baron MH. Inhibition of an erythroid differentiation switch by the helix-loop-helix protein Id1. J Biol Chem (1995) 270(30):17939–46. doi: 10.1074/jbc.270.30.17939 7629100

[26] Shoji W Yamamoto T Obinata M. The helix-loop-helix protein id inhibits differentiation of murine erythroleukemia cells. J Biol Chem (1994) 269(7):5078–84. doi: 10.1016/S0021-9258(17)37657-3 8106486

[27] Kreider BL Benezra R Rovera G Kadesch T. Inhibition of myeloid differentiation by the helix-loop-helix protein id. Science (1992) 255(5052):1700–2. doi: 10.1126/science.1372755 1372755

[28] Quesenberry PJ Iscove NN Cooper C Brady G Newburger PE Stein GS. Expression of basic helix-loop-helix transcription factors in explant hematopoietic progenitors. J Cell Biochem (1996) 61(3):478–88. doi: 10.1002/(SICI)1097-4644(19960601)61:3<478::AID-JCB15>3.0.CO;2-F 8761952

[29] Cooper CL Brady G Bilia F Iscove NN Quesenberry PJ. Expression of the id family helix-loop-helix regulators during growth and development in the hematopoietic system. Blood (1997) 89(9):3155–65. doi: 10.1182/blood.V89.9.3155 9129018

[30] Cooper CL Newburger PE. Differential expression of id genes in multipotent myeloid progenitor cells: Id-1 is induced by early-and late-acting cytokines while id-2 is selectively induced by cytokines that drive terminal granulocytic differentiation. J Cell Biochem (1998) 71(2):277–85. doi: 10.1002/(SICI)1097-4644(19981101)71:2<277::AID-JCB12>3.0.CO;2-I 9779825

[31] Jen Y Weintraub H Benezra R. Overexpression of id protein inhibits the muscle differentiation program: *in vivo* association of id with E2A proteins. Genes Dev (1992) 6(8):1466–79. doi: 10.1101/gad.6.8.1466 1644289

[32] Desprez PY Hara E Bissell MJ Campisi J. Suppression of mammary epithelial cell differentiation by the helix-loop-helix protein id-1. Mol Cell Biol (1995) 15(6):3398–404. doi: 10.1128/MCB.15.6.3398 PMC230574 7760836

[33] Leeanansaksiri W Wang H Gooya JM Renn K Abshari M Tsai S. IL-3 induces inhibitor of DNA-binding protein-1 in hemopoietic progenitor cells and promotes myeloid cell development. J Immunol (2005) 174(11):7014–21. doi: 10.4049/jimmunol.174.11.7014 15905544

[34] Cochrane SW Zhao Y Welner RS Sun XH. Balance between id and e proteins regulates myeloid-versus-lymphoid lineage decisions. Blood (2009) 113(5):1016–26. doi: 10.1182/blood-2008-06-164996 PMC2635070 18927439

[35] Jankovic V Ciarrocchi A Boccuni P DeBlasio T Benezra R Nimer SD. Id1 restrains myeloid commitment, maintaining the self-renewal capacity of hematopoietic stem cells. Proc Natl Acad Sci USA (2007) 104(4):1260–5. doi: 10.1073/pnas.0607894104 PMC1783103 17227850

[36] Singh SK Singh S Gadomski S Sun L Pfannenstein A Magidson V. Id1 ablation protects hematopoietic stem cells from stress-induced exhaustion and aging. Cell Stem Cell (2018) 23(2):252–65.e8. doi: 10.1016/j.stem.2018.06.001 30082068 PMC6149219

[37] Perry SS Zhao Y Nie L Cochrane SW Huang Z Sun XH. Id1, but not Id3, directs long-term repopulating hematopoietic stem-cell maintenance. Blood (2007) 110(7):2351–60. doi: 10.1182/blood-2007-01-069914 PMC1988946 17622570

[38] van Galen P Kreso A Wienholds E Laurenti E Eppert K Lechman ER. Reduced lymphoid lineage priming promotes human hematopoietic stem cell expansion. Cell Stem Cell (2014) 14(1):94–106. doi: 10.1016/j.stem.2013.11.021 24388174

[39] Suh HC Leeanansaksiri W Ji M Klarmann KD Renn K Gooya J. Id1 immortalizes hematopoietic progenitors *in vitro* and promotes a myeloproliferative disease *in vivo* . Oncogene (2008) 27(42):5612–23. doi: 10.1038/onc.2008.175 PMC3073486 18542061

[40] Buitenhuis M van Deutekom HW Verhagen LP Castor A Jacobsen SE Lammers JW. Differential regulation of granulopoiesis by the basic helix-loop-helix transcriptional inhibitors Id1 and Id2. Blood (2005) 105(11):4272–81. doi: 10.1182/blood-2004-12-4883 15701714

[41] Morrow MA Mayer EW Perez CA Adlam M Siu G. Overexpression of the helix-Loop-Helix protein Id2 blocks T cell development at multiple stages. Mol Immunol (1999) 36(8):491–503. doi: 10.1016/S0161-5890(99)00071-1 10475604

[42] Kim D Peng XC Sun XH. Massive apoptosis of thymocytes in T-cell-deficient Id1 transgenic mice. Mol Cell Biol (1999) 19(12):8240–53. doi: 10.1128/MCB.19.12.8240 PMC84908 10567549

[43] Sun XH. Constitutive expression of the Id1 gene impairs mouse b cell development. Cell (1994) 79(5):893–900. doi: 10.1016/0092-8674(94)90078-7 8001126

[44] Belle I Zhuang Y. E proteins in lymphocyte development and lymphoid diseases. Curr topics Dev Biol (2014) 110:153–87. doi: 10.1016/B978-0-12-405943-6.00004-X PMC6504980 25248476

[45] Mercer EM Lin YC Benner C Jhunjhunwala S Dutkowski J Flores M. Multilineage priming of enhancer repertoires precedes commitment to the b and myeloid cell lineages in hematopoietic progenitors. Immunity (2011) 35(3):413–25. doi: 10.1016/j.immuni.2011.06.013 PMC3183365 21903424

[46] Mercer EM Lin YC Murre C. Factors and networks that underpin early hematopoiesis. Semin Immunol (2011) 23(5):317–25. doi: 10.1016/j.smim.2011.08.004 PMC3217090 21930392

[47] Ikawa T Masuda K Huijskens M Satoh R Kakugawa K Agata Y. Induced developmental arrest of early hematopoietic progenitors leads to the generation of leukocyte stem cells. Stem Cell Rep (2015) 5(5):716–27. doi: 10.1016/j.stemcr.2015.09.012 PMC4649263 26607950

[48] Kee BL Rivera RR Murre C. Id3 inhibits b lymphocyte progenitor growth and survival in response to TGF-beta. Nat Immunol (2001) 2(3):242–7. doi: 10.1038/85303 11224524

[49] Pan L Sato S Frederick JP Sun XH Zhuang Y. Impaired immune responses and b-cell proliferation in mice lacking the Id3 gene. Mol Cell Biol (1999) 19(9):5969–80. doi: 10.1128/MCB.19.9.5969 PMC84466 10454544

[50] Rivera RR Johns CP Quan J Johnson RS Murre C. Thymocyte selection is regulated by the helix-loop-helix inhibitor protein, Id3. Immunity (2000) 12(1):17–26. doi: 10.1016/S1074-7613(00)80155-7 10661402

[51] Thal MA Carvalho TL He T Kim HG Gao H Hagman J. Ebf1-mediated down-regulation of Id2 and Id3 is essential for specification of the b cell lineage. Proc Natl Acad Sci USA (2009) 106(2):552–7. doi: 10.1073/pnas.0802550106 PMC2626741 19122139

[52] Yan W Young AZ Soares VC Kelley R Benezra R Zhuang Y. High incidence of T-cell tumors in E2A-null mice and E2A/Id1 double-knockout mice. Mol Cell Biol (1997) 17(12):7317–27. doi: 10.1128/MCB.17.12.7317 PMC232588 9372963

[53] Suh HC Ji M Gooya J Lee M Klarmann KD Keller JR. Cell-nonautonomous function of Id1 in the hematopoietic progenitor cell niche. Blood (2009) 114(6):1186–95. doi: 10.1182/blood-2008-09-179788 PMC2723014 19478045

[54] Yang J Li X Morrell NW. Id proteins in the vasculature: from molecular biology to cardiopulmonary medicine. Cardiovasc Res (2014) 104(3):388–98. doi: 10.1093/cvr/cvu215 25274246

[55] Lyden D Young AZ Zagzag D Yan W Gerald W O’Reilly R. Id1 and Id3 are required for neurogenesis, angiogenesis and vascularization of tumour xenografts. Nature (1999) 401(6754):670–7. doi: 10.1038/44334 10537105

[56] Gadomski S Singh SK Singh S Sarkar T Klarmann KD Berenschot M. Id1 and Id3 maintain steady-state hematopoiesis by promoting sinusoidal endothelial cell survival and regeneration. Cell Rep (2020) 31(4):107572. doi: 10.1016/j.celrep.2020.107572 32348770 PMC8459380

[57] Peng Y Kang Q Luo Q Jiang W Si W Liu BA. Inhibitor of DNA binding/differentiation helix-loop-helix proteins mediate bone morphogenetic protein-induced osteoblast differentiation of mesenchymal stem cells. J Biol Chem (2004) 279(31):32941–9. doi: 10.1074/jbc.M403344200 15161906

[58] Fraidenraich D Stillwell E Romero E Wilkes D Manova K Basson CT. Rescue of cardiac defects in id knockout embryos by injection of embryonic stem cells. Science (2004) 306(5694):247–52. doi: 10.1126/science.1102612 PMC1351017 15472070

[59] Morrison SJ Scadden DT. The bone marrow niche for haematopoietic stem cells. Nature (2014) 505(7483):327–34. doi: 10.1038/nature12984 PMC4514480 24429631

[60] Wei Q Frenette PS. Niches for hematopoietic stem cells and their progeny. Immunity (2018) 48(4):632–48. doi: 10.1016/j.immuni.2018.03.024 PMC6103525 29669248

[61] Kusumbe AP Ramasamy SK Itkin T Mae MA Langen UH Betsholtz C. Age-dependent modulation of vascular niches for haematopoietic stem cells. Nature (2016) 532(7599):380–4. doi: 10.1038/nature17638 PMC5035541 27074508

[62] Itkin T Gur-Cohen S Spencer JA Schajnovitz A Ramasamy SK Kusumbe AP. Distinct bone marrow blood vessels differentially regulate haematopoiesis. Nature (2016) 532(7599):323–8. doi: 10.1038/nature17624 PMC6450701 27074509

[63] Comazzetto S Shen B Morrison SJ. Niches that regulate stem cells and hematopoiesis in adult bone marrow. Dev Cell (2021) 56(13):1848–60. doi: 10.1016/j.devcel.2021.05.018 PMC8282762 34146467

[64] Rafii S Butler JM Ding BS. Angiocrine functions of organ-specific endothelial cells. Nature (2016) 529(7586):316–25. doi: 10.1038/nature17040 PMC4878406 26791722

[65] Kobayashi H Butler JM O’Donnell R Kobayashi M Ding BS Bonner B. Angiocrine factors from akt-activated endothelial cells balance self-renewal and differentiation of haematopoietic stem cells. Nat Cell Biol (2010) 12(11):1046–56. doi: 10.1038/ncb2108 PMC2972406 20972423

[66] Kusumbe AP Ramasamy SK Adams RH. Coupling of angiogenesis and osteogenesis by a specific vessel subtype in bone. Nature (2014) 507(7492):323–8. doi: 10.1038/nature13145 PMC4943525 24646994

[67] Ding BS Cao Z Lis R Nolan DJ Guo P Simons M. Divergent angiocrine signals from vascular niche balance liver regeneration and fibrosis. Nature (2014) 505(7481):97–102. doi: 10.1038/nature12681 24256728 PMC4142699

[68] Ding BS Nolan DJ Butler JM James D Babazadeh AO Rosenwaks Z. Inductive angiocrine signals from sinusoidal endothelium are required for liver regeneration. Nature (2010) 468(7321):310–5. doi: 10.1038/nature09493 PMC3058628 21068842

[69] Poulos MG Guo P Kofler NM Pinho S Gutkin MC Tikhonova A. Endothelial jagged-1 is necessary for homeostatic and regenerative hematopoiesis. Cell Rep (2013) 4(5):1022–34. doi: 10.1016/j.celrep.2013.07.048 PMC3805263 24012753

[70] Kfoury Y Scadden DT. Mesenchymal cell contributions to the stem cell niche. Cell Stem Cell (2015) 16(3):239–53. doi: 10.1016/j.stem.2015.02.019 25748931

[71] Li H Dai M Zhuang Y. A T cell intrinsic role of Id3 in a mouse model for primary sjogren’s syndrome. Immunity (2004) 21(4):551–60. doi: 10.1016/j.immuni.2004.08.013 15485632

[72] Li J Maruyama T Zhang P Konkel JE Hoffman V Zamarron B. Mutation of inhibitory helix-loop-helix protein Id3 causes gammadelta T-cell lymphoma in mice. Blood (2010) 116(25):5615–21. doi: 10.1182/blood-2010-03-274506 PMC3031408 20852128

[73] Ji M Li H Suh HC Klarmann KD Yokota Y Keller JR. Id2 intrinsically regulates lymphoid and erythroid development *via* interaction with different target proteins. Blood (2008) 112(4):1068–77. doi: 10.1182/blood-2008-01-133504 PMC2515127 18523151

[74] Hock H Hamblen MJ Rooke HM Schindler JW Saleque S Fujiwara Y. Gfi-1 restricts proliferation and preserves functional integrity of haematopoietic stem cells. Nature (2004) 431(7011):1002–7. doi: 10.1038/nature02994 15457180

[75] Zeng H Yucel R Kosan C Klein-Hitpass L Moroy T. Transcription factor Gfi1 regulates self-renewal and engraftment of hematopoietic stem cells. EMBO J (2004) 23(20):4116–25. doi: 10.1038/sj.emboj.7600419 PMC524350 15385956

[76] Li H Ji M Klarmann KD Keller JR. Repression of Id2 expression by gfi-1 is required for b-cell and myeloid development. Blood (2010) 116(7):1060–9. doi: 10.1182/blood-2009-11-255075 PMC2938128 20453161

[77] Kim W Klarmann KD Keller JR. Gfi-1 regulates the erythroid transcription factor network through Id2 repression in murine hematopoietic progenitor cells. Blood (2014) 124(10):1586–96. doi: 10.1182/blood-2014-02-556522 PMC4155270 25051963

[78] Jakubison BL Sarkar T Gudmundsson KO Singh S Sun L Morris HM. ID2 and HIF-1α collaborate to protect quiescent hematopoietic stem cells from activation, differentiation, and exhaustion. J Clin Invest (2022) 132(13). doi: 10.1172/JCI152599 PMC9246389 35775482

[79] Takubo K Goda N Yamada W Iriuchishima H Ikeda E Kubota Y. Regulation of the HIF-1alpha level is essential for hematopoietic stem cells. Cell Stem Cell (2010) 7(3):391–402. doi: 10.1016/j.stem.2010.06.020 20804974

[80] Barone MV Pepperkok R Peverali FA Philipson L. Id proteins control growth induction in mammalian cells. Proc Natl Acad Sci USA (1994) 91(11):4985–8. doi: 10.1073/pnas.91.11.4985 PMC43914 8197168

[81] Ganuza M McKinney-Freeman S. Hematopoietic stem cells under pressure. Curr Opin Hematol (2017) 24(4):314–21. doi: 10.1097/MOH.0000000000000347 PMC5669070 28375987

[82] Termini CM Chute JP. Hematopoietic stem cell stress and regeneration. Curr Stem Cell Rep (2020) 6(4):134–43. doi: 10.1007/s40778-020-00181-3

[83] Boettcher S Manz MG. Regulation of inflammation- and infection-driven hematopoiesis. Trends Immunol (2017) 38(5):345–57. doi: 10.1016/j.it.2017.01.004 28216309

[84] Caiado F Pietras EM Manz MG. Inflammation as a regulator of hematopoietic stem cell function in disease, aging, and clonal selection. J Exp Med (2021) 218(7). doi: 10.1084/jem.20201541 PMC8210622 34129016

[85] Singh S Jakubison B Keller JR. Protection of hematopoietic stem cells from stress-induced exhaustion and aging. Curr Opin Hematol (2020) 27(4):225–31. doi: 10.1097/MOH.0000000000000586 32398455

[86] Kleppe M Spitzer MH Li S Hill CE Dong L Papalexi E. Jak1 integrates cytokine sensing to regulate hematopoietic stem cell function and stress hematopoiesis. Cell Stem Cell (2018) 22(2):277. doi: 10.1016/j.stem.2017.12.018 29395057 PMC6260818

[87] Zhao Y Ling F Wang HC Sun XH. Chronic TLR signaling impairs the long-term repopulating potential of hematopoietic stem cells of wild type but not Id1 deficient mice. PloS One (2013) 8(2):e55552. doi: 10.1371/journal.pone.0055552 23383338 PMC3562238

[88] Barreyro L Chlon TM Starczynowski DT. Chronic immune response dysregulation in MDS pathogenesis. Blood (2018) 132(15):1553–60. doi: 10.1182/blood-2018-03-784116 PMC6182269 30104218

[89] Trowbridge JJ Starczynowski DT. Innate immune pathways and inflammation in hematopoietic aging, clonal hematopoiesis, and MDS. J Exp Med (2021) 218(7). doi: 10.1084/jem.20201544 PMC8210621 34129017

[90] Craver BM El Alaoui K Scherber RM Fleischman AG. The critical role of inflammation in the pathogenesis and progression of myeloid malignancies. Cancers (Basel) (2018) 10(4):104–20. 10.3390/cancers10040104 PMC5923359 29614027

[91] Prabhu S Ignatova A Park ST Sun XH. Regulation of the expression of cyclin-dependent kinase inhibitor p21 by E2A and id proteins. Mol Cell Biol (1997) 17(10):5888–96. 10.1128/mcb.17.10.5888 PMC232436 9315646

[92] Rothschild G Zhao X Iavarone A Lasorella A. E proteins and Id2 converge on p57Kip2 to regulate cell cycle in neural cells. Mol Cell Biol (2006) 26(11):4351–61. doi: 10.1128/MCB.01743-05 PMC1489106 16705184

[93] Mern DS Hoppe-Seyler K Hoppe-Seyler F Hasskarl J Burwinkel B. Targeting Id1 and Id3 by a specific peptide aptamer induces e-box promoter activity, cell cycle arrest, and apoptosis in breast cancer cells. Breast Cancer Res Treat (2010) 124(3):623–33. doi: 10.1007/s10549-010-0810-6 20191379

[94] Zheng W Wang H Xue L Zhang Z Tong T. Regulation of cellular senescence and p16(INK4a) expression by Id1 and E47 proteins in human diploid fibroblast. J Biol Chem (2004) 279(30):31524–32. doi: 10.1074/jbc.M400365200 15138269

[95] Semerad CL Mercer EM Inlay MA Weissman IL Murre C. E2A proteins maintain the hematopoietic stem cell pool and promote the maturation of myelolymphoid and myeloerythroid progenitors. Proc Natl Acad Sci USA (2009) 106(6):1930–5. doi: 10.1073/pnas.0808866106 PMC2644141 19181846

[96] Yang Q Esplin B Borghesi L. E47 regulates hematopoietic stem cell proliferation and energetics but not myeloid lineage restriction. Blood (2011) 117(13):3529–38. doi: 10.1182/blood-2010-07-297689 PMC3072876 21273306

[97] Dias S Mansson R Gurbuxani S Sigvardsson M Kee BL. E2A proteins promote development of lymphoid-primed multipotent progenitors. Immunity (2008) 29(2):217–27. doi: 10.1016/j.immuni.2008.05.015 PMC2600583 18674933

[98] Cheng T Rodrigues N Shen H Yang Y Dombkowski D Sykes M. Hematopoietic stem cell quiescence maintained by p21cip1/waf1. Science (2000) 287(5459):1804–8. doi: 10.1126/science.287.5459.1804 10710306

[99] Santos PM Ding Y Borghesi L. Cell-intrinsic *in vivo* requirement for the E47-p21 pathway in long-term hematopoietic stem cells. J Immunol (2014) 192(1):160–8. doi: 10.4049/jimmunol.1302502 PMC3893818 24259504

[100] Nair R Teo WS Mittal V Swarbrick A. ID Proteins regulate diverse aspects of cancer progression and provide novel therapeutic opportunities. Mol Ther: J Am Soc Gene Ther (2014) 22(8):1407–15. doi: 10.1038/mt.2014.83 PMC4435600 24827908

[101] Radich JP Dai H Mao M Oehler V Schelter J Druker B. Gene expression changes associated with progression and response in chronic myeloid leukemia. Proc Natl Acad Sci USA (2006) 103(8):2794–9. doi: 10.1073/pnas.0510423103 PMC1413797 16477019

[102] Rucker FG Bullinger L Schwaenen C Lipka DB Wessendorf S Frohling S. Disclosure of candidate genes in acute myeloid leukemia with complex karyotypes using microarray-based molecular characterization. J Clin Oncol (2006) 24(24):3887–94. doi: 10.1200/JCO.2005.04.5450 16864856

[103] Zhou JD Ma JC Zhang TJ Li XX Zhang W Wu DH. High bone marrow ID2 expression predicts poor chemotherapy response and prognosis in acute myeloid leukemia. Oncotarget (2017) 8(54):91979–89. doi: 10.18632/oncotarget.20559 PMC5696157 29190891

[104] Zhou JD Yang L Zhu XW Wen XM Yang J Guo H. Clinical significance of up-regulated ID1 expression in Chinese *de novo* acute myeloid leukemia. Int J Clin Exp Pathol (2015) 8(5):5336–44. PMC4503106 26191235

[105] Tochareontanaphol C Sinthuwiwat T Buathong B Thita T Promso S Paca-Uccaralertkun S. New mutations of the id1 gene in acute myeloid leukemia patients. Pathobiology (2015) 82(1):43–7. doi: 10.1159/000370243 25766257

[106] Klarmann K Ji M Li H Satyanarayana A Kim W Bowers E Novel targets in myelogenous leukemia: the id family of proteins. myeloid leukemia – basic mechanisms of leukemogenesis. Koschmieder S Krug U editors. Rijeka, Croatia: InTech (2011). 215–38 p.

[107] Bera R Chiu MC Huang YJ Lin TH Kuo MC Shih LY. RUNX1 mutations promote leukemogenesis of myeloid malignancies in ASXL1-mutated leukemia. J Hematol Oncol (2019) 12(1):104. doi: 10.1186/s13045-019-0789-3 31640815 PMC6805634

[108] Liquori A Ibanez M Sargas C Sanz MA Barragan E Cervera J. Acute promyelocytic leukemia: A constellation of molecular events around a single pml-rara fusion gene. Cancers (Basel) (2020) 12(3):624–6. doi: 10.3390/cancers12030624 PMC7139833 32182684

[109] Sayar H Liu Y Gao R Zaid MA Cripe LD Weisenbach J. Consecutive epigenetically-active agent combinations act in ID1-RUNX3-TET2 and HOXA pathways for Flt3ITD+ve AML. Oncotarget (2018) 9(5):5703–15. doi: 10.18632/oncotarget.23655 PMC5814168 29464028

[110] Eisfeld AK Kohlschmidt J Mrozek K Volinia S Blachly JS Nicolet D. Mutational landscape and gene expression patterns in adult acute myeloid leukemias with monosomy 7 as a sole abnormality. Cancer Res (2017) 77(1):207–18. doi: 10.1158/0008-5472.CAN-16-1386 PMC5215102 27784745

[111] Valk PJ Verhaak RG Beijen MA Erpelinck CA Barjesteh van Waalwijk van Doorn-Khosrovani S Boer JM. Prognostically useful gene-expression profiles in acute myeloid leukemia. N Engl J Med (2004) 350(16):1617–28. doi: 10.1056/NEJMoa040465 15084694

[112] Tam WF Gu TL Chen J Lee BH Bullinger L Frohling S. Id1 is a common downstream target of oncogenic tyrosine kinases in leukemic cells. Blood (2008) 112(5):1981–92. doi: 10.1182/blood-2007-07-103010 PMC2518899 18559972

[113] Tang R Hirsch P Fava F Lapusan S Marzac C Teyssandier I. High Id1 expression is associated with poor prognosis in 237 patients with acute myeloid leukemia. Blood (2009) 114(14):2993–3000. doi: 10.1182/blood-2009-05-223115 19643984

[114] Wang L Gural A Sun XJ Zhao X Perna F Huang G. The leukemogenicity of AML1-ETO is dependent on site-specific lysine acetylation. Science (2011) 333(6043):765–9. doi: 10.1126/science.1201662 PMC3251012 21764752

[115] Wang L Man N Sun XJ Tan Y Garcia-Cao M Liu F. Regulation of AKT signaling by Id1 controls t (8,21)Leukemia initiation and progression. Blood (2015) 126(5):640–50. doi: 10.1182/blood-2015-03-635532 PMC4520879 26084673

[116] Man N Sun XJ Tan Y Garcia-Cao M Liu F Cheng G. Differential role of Id1 in MLL-AF9-driven leukemia based on cell of origin. Blood (2016) 127(19):2322–6. doi: 10.1182/blood-2015-11-677708 PMC4865589 26944543

[117] Wojnarowicz PM Escolano MG Huang YH Desai B Chin Y Shah R. Anti-tumor effects of an ID antagonist with no observed acquired resistance. NPJ Breast Cancer (2021) 7(1):58. doi: 10.1038/s41523-021-00266-0 34031428 PMC8144414

[118] Soroceanu L Murase R Limbad C Singer E Allison J Adrados I. Id-1 is a key transcriptional regulator of glioblastoma aggressiveness and a novel therapeutic target. Cancer Res (2013) 73(5):1559–69. doi: 10.1158/0008-5472.CAN-12-1943 PMC3594064 23243024

[119] McAllister SD Christian RT Horowitz MP Garcia A Desprez PY. Cannabidiol as a novel inhibitor of id-1 gene expression in aggressive breast cancer cells. Mol Cancer Ther (2007) 6(11):2921–7. doi: 10.1158/1535-7163.MCT-07-0371 18025276

[120] Kovtonyuk LV Fritsch K Feng X Manz MG Takizawa H. Inflamm-aging of hematopoiesis, hematopoietic stem cells, and the bone marrow microenvironment. Front Immunol (2016) 7:502. doi: 10.3389/fimmu.2016.00502 27895645 PMC5107568

[121] Ko J Patel N Ikawa T Kawamoto H Frank O Rivera RR. Suppression of e-protein activity interferes with the development of BCR-ABL-mediated myeloproliferative disease. Proc Natl Acad Sci U S A (2008) 105(35):12967–72. doi: 10.1073/pnas.0805073105 PMC2529058 18725623

[122] Ghisi M Kats L Masson F Li J Kratina T Vidacs E. Id2 and e proteins orchestrate the initiation and maintenance of MLL-rearranged acute myeloid leukemia. Cancer Cell (2016) 30(1):59–74. doi: 10.1016/j.ccell.2016.05.019 27374225

[123] Nigmatullina L Norkin M Dzama MM Messner B Sayols S Soshnikova N. Id2 controls specification of Lgr5(+) intestinal stem cell progenitors during gut development. EMBO J (2017) 36(7):869–85. doi: 10.15252/embj.201694959 PMC5376969 28077488

[124] Russell RG Lasorella A Dettin LE Iavarone A. Id2 drives differentiation and suppresses tumor formation in the intestinal epithelium. Cancer Res (2004) 64(20):7220–5. doi: 10.1158/0008-5472.CAN-04-2095 15492237

